# Neoadjuvant radiotherapy combined with fluorouracil-cisplatin plus cetuximab in operable, locally advanced esophageal carcinoma: Results of a phase I-II trial (FFCD-0505/PRODIGE-3)

**DOI:** 10.1016/j.ctro.2024.100804

**Published:** 2024-06-09

**Authors:** Bernadette de Rauglaudre, Guillaume Piessen, Marine Jary, Karine Le Malicot, Antoine Adenis, Thibault Mazard, Xavier Benoît D’Journo, Caroline Petorin, Joelle Buffet-Miny, Thomas Aparicio, Rosine Guimbaud, Véronique Vendrely, Côme Lepage, Laetitia Dahan

**Affiliations:** a Department of Digestive Oncology, Hôpital la Timone, Marseille, France; b Department of Digestive Surgery, University Hospital Huriez, Lille, France; c Department of Digestive Surgery, University Hospital Estaing, Clermont-Ferrand, France; d Fédération Francophone de Cancérologie Digestive (FFCD), EPICAD INSERM LNC-UMR 1231, University of Burgundy and Franche-Comté, Dijon, France; e Department of Medical Oncology, Centre Oscar Lambret, Lille, France; f Department of Medical Oncology, ICM Val d’Aurelle, Montpellier, France; g Department of Thoracic Surgery, Aix-Marseille University, Hôpital Nord, Marseille, France; h Department of Radiotherapy, University Hospital Jean Minjoz, Besançon, France; i Department of Digestive Oncology, Hôpital Saint-Louis, Paris, France; j Department of Medical Oncology, CHU de Toulouse, Toulouse, France; k Department of Radiotherapy, Hôpital Saint-André, Bordeaux, France; l Department of Digestive Oncology, Hôpital François Mitterrand, Dijon, France

**Keywords:** Cetuximab, Cisplatin, Esophageal carcinoma, Fluorouracil, Locally advanced, Neoadjuvant, Phase I/II trial, Radiochemotherapy

## Abstract

**Background:**

Radiotherapy combined with fluorouracil (5FU) and cisplatin for locally advanced esophageal cancer is associated with a 20–25% pathologic complete response (pCR) rate. Cetuximab increases the efficacy of radiotherapy in patients with head and neck carcinomas. The aim of this phase I/II trial was to determine the optimal doses and the pCR rate with chemoradiotherapy (C-RT) plus cetuximab.

**Methods:**

A 45-Gy radiotherapy regimen was delivered over 5 weeks. The phase I study determined the dose-limiting toxicity and the maximum tolerated dose of 5FU-cisplatin plus cetuximab. The phase II trial aimed to exhibit a pCR rate > 20 % (25 % expected), requiring 33 patients (6 from phase I part plus 27 in phase II part). pCR was defined as ypT0Nx.

**Results:**

The phase I study established the following recommended doses: weekly cetuximab (400 mg/m^2^ one week before, and 250 mg/m^2^ during radiotherapy); 5FU (500 mg/m^2^/day, d1-d4) plus cisplatin (40 mg/m^2^, d1) during week 1 and 5. In the phase II part, 32 patients received C-RT before surgery, 31 patients underwent surgery, and resection was achieved in 27 patients. A pCR was achieved in five patients (18.5 %) out of 27. After a median follow-up of 19 months, the median progression-free survival was 13.7 months, and the median overall survival was not reached.

**Conclusions:**

Adding cetuximab to preoperative C-RT was toxic and did not achieve a pCR > 20 % as required. The recommended doses, determined during the phase I part, could explain these disappointing results due to a reduction in chemotherapy dose-intensity.

**Trial registration:**

This trial was registered with EudraCT number 2006-004770-27.

## Highlights

• Adding Cetuximab to chemoradiotherapy in advanced esophageal cancer could improve pCR.
• With a 5 weeks radiotherapy, we determined the optimal doses for 5FU, Cisplatin, Cetuximab based chemotherapy.
• This regimen failed to reach a pCR > 20 %, and showed to be toxic.

## Background

The diagnosis of esophageal cancer is often late because of the patients’ profile. Therefore, only 50 % of patients with newly diagnosed esophageal carcinoma are suitable candidates for surgery. As most of the operated patients relapse and die, preoperative approaches have been developed for decades [1], [2], [3], [4], [5]. Preoperative chemoradiotherapy (C-RT) has been shown to provide a survival benefit over surgery alone, without revealing the optimal C-RT regimen to use [2], [3], [6]. However, this benefit was not found in all studies [4], [7]. Among published trials evaluating neoadjuvant C-RT, none has been helpful in regimen decision-making [3], [4], [8]. For instance, in the FFCD-9901 trial evaluating a preoperative cisplatin (CDDP)-fluorouracil (5FU) doublet combined with 45-Gy radiotherapy (RT) no survival benefit over surgery alone was found [4]. On the other hand, a meaningful survival benefit was reported in the CROSS study, favoring a weekly administration of carboplatin-paclitaxel combined with a concurrent RT of 41.4 Gy followed by surgery, compared with surgery alone [3]. A comparison of these regimens has been published in the PROSPECT trial, showing similar efficacy between an oxaliplatine-5FU doublet and carboplatin-paclitaxel combined with a concurrent RT [5]. However, carboplatin-paclitaxel was associated with a higher-than-expected rate of severe postoperative morbidity [9].

To date, the best preoperative regimen is still to be determined, and new active cytotoxics or targeted therapies are awaited. Targeting the epidermal growth factor receptor (EGFR) pathway could be an option to investigate. In esophageal cancer, the EGFR is overexpressed in 72 % of SCC, and in 75 % of adenocarcinoma [10]. Moreover, adding cetuximab to neoadjuvant C-RT significantly improved loco-regional control and led to clinically relevant, but not significant, improvements in survival in the SAKK 75/08 trial of resectable esophageal carcinoma [11].

The aim of this phase I/II study, the PRODIGE-3 trial, was to determine dose-limiting toxicity (DLT) and recommended doses (RD) of neoadjuvant 5FU-CDDP plus cetuximab combined with RT, and to assess pathologic complete response (pCR) rate and safety profile of this combination when delivered at RD.

## Methods

### Study population

Previously untreated patients with locally advanced (Us T1N+, Us T2N0, Us T2N+, Us T3N0, Us T3N + ), operable, and histologically confirmed SCC or adenocarcinoma of the thoracic esophagus and gastroesophageal junction (Siewert I only) were eligible for this prospective multicenter trial. The main eligibility criteria were age ≥ 18 years; World Health Organization performance status (WHO-PS) < 2; weight loss < 15 %; no cirrhosis; adequate hematologic, liver, and renal functions; and no respiratory insufficiency.

Exclusion criteria included total esophagostomy without thoracotomy, palliative surgery, Siewert II-III cervical esophagus carcinoma or adenocarcinoma of the gastroesophageal junction, previous chemotherapy or RT, and previous esophagus stent.

Written informed consent was obtained before inclusion. The protocol was reviewed and approved by the Ethics Committee (Sud-Mediterranée II, Marseille, December 1st 2006) and the study was conducted according to the Declaration of Helsinki and European Good Clinical Practice requirements.

The pretherapeutic evaluation included medical history, physical examination, hematological and biochemical assessments, upper endoscopy plus biopsy, endoscopic ultrasonography, thoraco-abdominal computed tomography (CT) scan, bronchoscopy, and optional positron emission tomography–computed tomography (PET-CT) scan.

### Neoadjuvant treatment

#### Radiotherapy

**Fig. 1:**
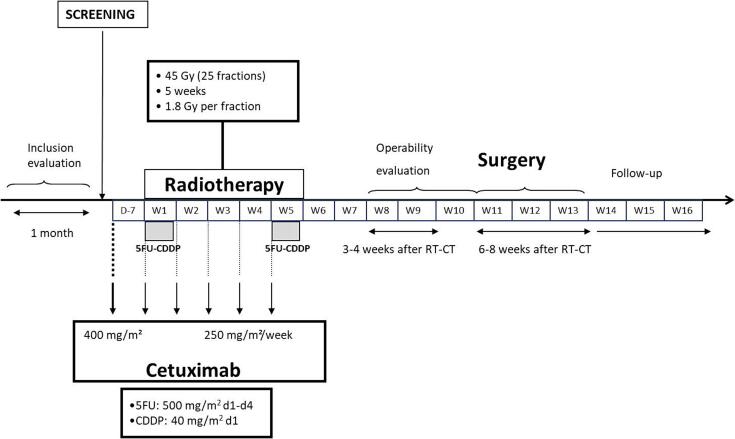
Treatment plan of PRODIGE-3 trial.

The RT started on the same day as the first chemotherapy dose, and was delivered for 5 weeks in 25 fractions of 1.8 Gy each, on 5 days per week (excluding weekends), for a total dose of 45 Gy (Fig. 1). Target volumes were determined by a pretherapeutic CT-scan at the time of enrolment, defining clinical (CTV) and planned target volumes (PTV), as well as adjacent organs. Target volumes were determined in accordance with ICRU (International Commission on Radiation Units and Measurements) recommendations. The PTV-1 corresponded to a multidirectional irradiation of one cm around the CTV, for a total dose of 39.6 Gy (homogeneity from + 7 % to −5%). The PTV-2 corresponded to a multidirectional irradiation of one cm around the gross tumor volume (GTV), for a total dose of 5.4 Gy.

RT was discontinued in cases of suspected respiratory fistula or mediastinitis, or rapid deterioration in general condition (WHO 3–4) with weight loss > 10 %.

#### Chemotherapy

In the phase I study, 5FU and CDDP were planned to be escalated according to 4 dose levels (level −1: 500/40 mg/m^2^; level 0: 600/60 mg/m^2^; level 1: 800/60 mg/m^2^; level 2: 800/75 mg/m^2^). During weeks 1 and 5 of RT, continuous infusion of 5FU was administered daily from day 1 to day 4, and continuous infusion of CDDP was delivered on day 1 (Fig. 1).

#### Cetuximab

Cetuximab dosing was constant across the different chemotherapy dose levels. A one-hour infusion of cetuximab was administered weekly at 400 mg/m^2^ one week before the onset of RT, and at 250 mg/m^2^ during RT (Fig. 1).

### Safety assessment

All toxicities were graded according to NCI-CTC criteria (v3.0). Serious adverse events were recorded within 24 h.

After inclusion, a complete clinical examination and full laboratory investigations were performed every week. Hemoglobin, platelet and white blood cells (WBC) count were performed weekly during RT.

After neoadjuvant treatment, physical examination, radiological assessment (thoraco-abdominal CT-scan), upper endoscopy, and respiratory evaluation were performed.

### Dose-limiting toxicity assessment

The DLT was defined as any grade 4 hematologic toxicity, grade 4 esophagitis, any non-hematologic severe toxicity (except dysphagia, nausea, vomiting, alopecia, grade 3 esophagitis and grade 3 acneiform reaction), and postoperative complications. The DLT was also defined by the dose level that did not allow delivering scheduled subsequent chemotherapy courses for any medical reason.

Three or six patients were treated at each dose level. If one of three patients at a given dose level developed DLT, a further three patients were entered at the same dose level. The maximal tolerated dose (MTD) was defined as the dose at which at least two of the first three patients or at least three of the six patients developed DLT. The recommended dose was defined as the dose level just below the MTD. Then, a phase II study was conducted according to the decision of an independent committee.

### Surgery

A restaging imaging and endoscopy was undertaken 4–6 weeks after C-RT. A curative surgery (*i.e.* clear margins [R0]) was performed 6 to 8 weeks after C-RT (Fig. 1). The surgical procedure was a single-stage operation, which combined a transthoracic en-bloc subtotal esophagectomy with a radical two-field lymphadenectomy. A gastric interposition in the posterior mediastinum was the preferred substitute for resected esophagus reconstruction. The anastomosis was located in the chest or in the neck, depending on the location of the tumor and the histopathologic subtype. For adenocarcinoma below the carina, an intrathoracic anastomosis was performed. For SCC or for long Barrett’s esophagus, a cervical anastomosis was preferred. The en-bloc esophagectomy with radical lymphadenectomy consisted in the resection of all *peri*-esophageal tissues in the mediastinum (including thoracic duct, azygos vein, ipsilateral mediastinal pleura, upper and lower mediastinal lymph nodes [LNs]), and in the abdomen (LNs of the hepatic artery, coeliac trunk, splenic artery and nodes at the basis of the left gastric artery). The surgeon labeled each LN according to its location, which were analyzed separately by the pathologist.

### Statistical methods

The primary endpoints of the phase I study were DLT and MTD. Patients' individual data were reviewed by an independent committee defining DLT and MTD. By using a Fibonacci method, at least nine patients were required.

In the phase II study, the primary endpoint was the short-term efficacy provided by the pCR rate based on Mandard criteria [12]. pCR was defined as ypT0Nx. Thirty-three patients were required on the basis of the following hypothesis: a 25 % increase in pCR rate was expected, a one-sided risk of 5 % (α), a power of 90 % (1 – β). The secondary endpoints were postoperative complications, resectability rate, relapse-free survival (RFS), overall survival (OS) and overall safety.

All assessable patients were included in an intention-to-treat (ITT) analysis using Stata statistical software (version 10.0). Descriptive statistics were used for baseline parameters, efficacy and safety criteria. Quantitative data were described using mean, standard deviation (SD), median, and interquartile range (IQR). Qualitative data were presented using percentages and 95 % confidence interval (95-CI). Time-to-event criteria (RFS and OS) were computed by Kaplan-Meier method.

## Results

### Phase I study

**Fig. 2:**
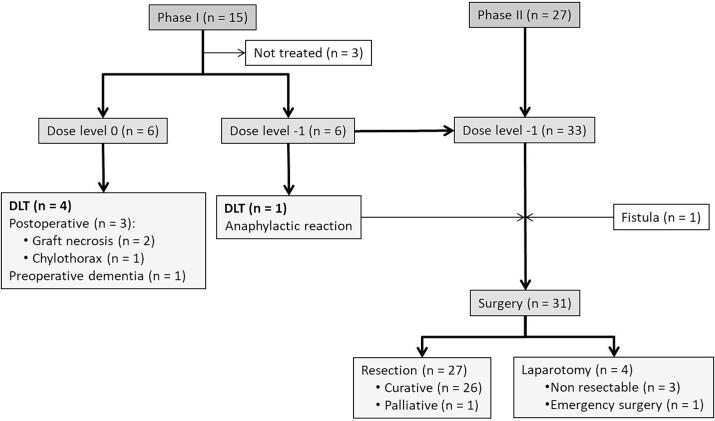
Study flow chart.

From July 2007 to June 2009, 12 patients were enrolled in the phase I study conducted across 7 centers. Six patients were enrolled at dose level 0, and six at dose level −1 (Fig. 2). Patients’ characteristics are summarized in Table 1.

**Table 1:** Baseline characteristics of the 33 enrolled patients.

Characteristics	Phase I (n = 12)	Phase II (n = 33)
Female, n (%)	2 (16.7)	5 (15.1)
WHO-PS		
0	7 (58.3)	21 (63.6)
1	5 (41.7)	12 (36.4)
Histology, n (%)		
SCC	6 (50.0)	20 (60.6)
Adenocarcinoma	6 (50.0)	13 (39.4)
Weight loss, n (%)		
< 15 %	11 (91.7)	31 (96.9)
≥ 15 %	1 (8.3)	1 (3.1)
Missing	−	1
Age (years), median (range)	55 (37–68)	60 (41–76)
Us T classification		
T1	2 (16.7)	2 (6.1)
T2	4 (33.3)	2 (6.1)
T3	6 (50.0)	29 (87.8)
Us N classification		
N0	1 (8.3)	4 (12.1)
N+	11 (91.7)	29 (87.9)

WHO-PS = World Health Organization performance status; SCC = squamous cell carcinoma.

Eleven patients (91.7 %) received the two cycles of chemotherapy, while one patient received only one cycle due to dementia. Additionally, one patient received only one infusion of cetuximab because of an anaphylactic reaction during the first week. All patients completed the five weeks of radiotherapy.

At dose level 0, four out of six patients presented with a DLT: one patient developed dementia before surgery, and three patients experienced postoperative complications (grade 4 chylothorax requiring subsequent surgery in one patient, graft necrosis requiring subsequent surgery in another and death due to graft necrosis in one patient). At dose level −1, one patient presented with a DLT due to a grade 4 cetuximab-related anaphylactic reaction. Toxicities are described in Table 2. Except the cetuximab-related anaphylactic reaction, no grade 3–4 toxicities occurred.

**Table 2:** Toxicities experienced during the phase I trial.

Toxicities	Level −1 (n = 6)	Level 0 (n = 6)
Preoperative, n	6	6
Allergy, n (%)		
Grade 0–2	5 (83.3)	6 (100)
Grade 3–4	1 (16.7)	0 (0)
Cutaneous, n (%)		
Grade 0–2	6 (100)	6 (100)
Grade 3–4	0 (0)	0 (0)
Hematologic, n (%)		
Grade 0–2	6 (100)	6 (100)
Grade 3–4	0 (0)	0 (0)
Esophagitis, n (%)		
Grade 0–2	6 (100)	4 (66.7)
Grade 3	0 (0)	2 (33.3)
Postoperative, n	5	6
Surgical, n (%)	2 (40.0)*	3 (50.0) ^†^
Medical, n (%)	1 (20.0) ^§^	3 (50.0) ^§^

* Anastomotic leak (n = 1), pleural complication plus gastroparesis (n = 1).

^†^ Anastomotic leak (n = 1), chylothorax (n = 1), graft necrosis (n = 1).

^§^ Acute respiratory distress syndrome.

The independent committee determined that the postoperative complications were unexpected in the context of standard esophageal surgery. Consequently, the RD for the phase II study was established as 5FU 500 mg/m^2^ (day 1 to day 4, week 1 and 5 of RT), CDDP 40 mg/m^2^ (day 1, week 1 and 5 of RT) and weekly cetuximab. Therefore, the RD level for phase II was determined to be weekly cetuximab (400 mg/m^2^ one week before the onset of RT, and 250 mg/m^2^ during RT).

### Phase II study

From February 2010 to January 2011, 27 patients were included in the phase II study conducted across 7 centers. In total, 33 patients were enrolled in the study: 6 from the phase I part at dose level −1, and 27 from phase II (Fig. 2). The patients’ characteristics are summarized in Table 1. One patient was not evaluable due to a fistula before the onset of treatment. Most of the patients were male, had a good WHO-PS and 60.6 % had SCC. The majority of patients were Us T3N + .

Thirty-one patients (94 %) received two cycles of chemotherapy (Table 3). Two patients received one cycle due to complications: one experienced gastric perforation during a gastrostomy procedure, and another had a catheter site injury. One patient received only one cetuximab infusion due to an anaphylactic reaction, as described in the phase I part.

**Table 3:** Neoadjuvant treatment delivery in 32 patients at dose level −1.

Treatment	n (%)
Chemotherapy	
One week	2 (6.2)
Two weeks	30 (93.8)
Cetuximab	
6 infusions	28 (87.5)
5 infusions	2 (6.3)
3 infusions	1 (3.1)
1 infusion	1 (3.1)
Radiotherapy	
25 fractions	30 (93.8)
28 fractions	1 (3.1)
7 fractions	1 (3.1)

### Surgery

Thirty-one patients underwent surgery, which included resections in 27 patients (87 %) and laparotomies without resection in 4 patients (three had non-resectable tumors, and one underwent unscheduled emergency surgery). One patient experienced disease progression before surgery. The surgical procedures consisted of transthoracic total esophagectomy with gastroesophageal anastomosis and lymphadenectomy of at least 15 nodes. Among the 27 patients who underwent esophagectomy, the resection was R0 in 25 patients (92.6 %), R1 in one patient, and palliative in one patient.

### Efficacy

A pCR was achieved in five patients (18.5 %) out of 27. Three patients were evaluated as pT0N0 (11.1 %). The pathological response is detailed in Table 4.

**Table 4:** Pathological response in 27 patients according to Mandard criteria [13].

Tumor regression grade	n (%)
1 = complete response	5 (18.5)
ypT0N0	3 (11.1)
ypT0N+	2 (7.4)
2 = residual cancer cells scattered through fibrosis	6 (22.2)
3 = increased number of residual cancer cells with predominant fibrosis	8 (29.6)
4 = residual cancer outgrowing fibrosis	6 (22.2)

After a median follow-up of 19.1 months (95-CI: 17.1 to 23.7 months), 18 patients (56.3 %) experienced relapse or died. The types of relapse were distant in 6 patients, local plus distant in 3 patients, and local in 4 patients. Five patients (15.6 %) died during the follow-up period, without relapse. The median RFS time was 13.7 months (95-CI: 5.7 months to “not reached”).

Overall, 13 patients died, 7 due to disease progression, and 6 from causes unrelated to esophageal carcinoma, including cardiorespiratory distress (1), multivisceral failure (2), esophageal fistula plus septic shock (1), acute respiratory distress leading to multivisceral failure (1), and pneumopathy (1). The median OS time was not reached (95-CI: 17.4 months to “not reached”).

### Safety

The distribution of the highest toxicities experienced during C-RT is presented in Table 5. One patient developed severe hematological toxicity. Cutaneous toxicity occurred in 78.1 % of patients, which one developing a grade 4 cetuximab-related rash. No preoperative treatment-related death occurred. Two patients discontinued C-RT prematurely due to gastric perforation (1) and cetuximab-related anaphylactic reaction (1).

**Table 5:** Higher toxicities experienced by 32 patients during chemoradiotherapy according to the NCI-CTC classification.

Toxicity*	Grade 1–2	Grade 3–4
Hematological		
Anemia	16 (50.0)	−
Lymphopenia	3 (9.4)	3 (9.4)
Leucopenia	13 (40.6)	1 (3.1)
Neutropenia	8 (25.0)	–
Thrombocytopenia	9 (28.1)	–

Cutaneous		
Acneiform reaction	27 (84.4)	1 (3.1)
Rash	14 (43.7)	−
Dryness	5 (15.6)	−
Nail injury	1 (3.1)	–
Infection	1 (3.1)	–

Gastrointestinal		
Anorexia	9 (28.1)	2 (6.3)
Diarrhea	2 (6.3)	−
Nausea	19 (59.4)	–
Vomiting	5 (15.6)	−
Stomatitis	13 (40.6)	–
Esophagitis	12 (37.5)	4 (12.5)
Dysphagia	8 (25.0)	3 (9.4)
Epigastralgia	7 (21.9)	−
Constipation	4 (12.5)	−
Gastroesophageal reflux	4 (12.5)	−
Perforation	–	1 (1.31)

Biological		
Hypoalbuminemia	6 (18.8)	–
Hypocalcemia	6 (18.8)	−
Hypokaliemia	1 (1.31)	–
Hyperkaliemia	−	1 (1.31)
Hyponatremia	1 (1.31)	−
Hyperglycemia	1 (1.31)	–
Hyperbilirubinemia	1 (1.31)	–

General symptoms		
Asthenia	17 (53.1)	2 (6.3)
Fever	3 (9.4)	–
Weight loss	3 (9.4)	−
Insomnia	2 (6.3)	−
Dysphonia	2 (6.3)	−
Cough	2 (6.3)	−
Dizziness	1 (1.31)	1 (1.31)

Other§	10 (31.2)	1 (3.1)

* Several toxicities could occur in a same patient. § Grade 1–2 other toxicities consisted of acroparesthesia (n = 1), cardiac disorder (n = 1), dyspnea (n = 2), ENT infection (n = 1), epistaxis (n = 2), gingival bleedings (n = 1), heavy legs (n = 1), and thoracic pains (n = 1); grade 3–4 toxicity consisted of supraclavicular vein thrombosis.

**Table 6:** Postoperative complications in 31 patients.

Complication	n (%)
Postoperative complications*	15 (48.4)
Surgical	8 (25.8)
Anastomotic leak	3 (9.7)
Anastomotic leak plus laryngeal paralysis	1 (3.2)
Chylothorax	1 (3.2)
Graft necrosis	1 (3.2)
Pleural complication	1 (3.2)
Pleural complication plus gastroparesis	1 (3.2)
Medical	13 (41.9)
Acute respiratory distress syndrome	8 (25.8)
Minor respiratory complications	4 (12.9)
Cardio-vascular complications	4 (12.9)
Neurological complications	3 (9.7)
Renal complications	2 (6.4)
Postoperative death	4 (12.9)
Clavien-Dindo score	
Grade 1	4 (12.9)
Grade 2a	3 (9.7)
Grade 2b	4 (12.9)
Grade 3	0 (0)
Grade 4	4 (12.9)

* Several postoperative complications could occur in a same patient.

Postoperative complication occurred in 15 out of 31 patients (48.4 %), AS presented in Table 6. Most of these postoperative adverse events were medical in nature (41.9 %). Eight patients developed acute respiratory distress syndrome (ARDS). Among surgical complications, two patients required repeated surgeries, one patient experienced an anastomotic leak, and one had a graft necrosis. There were four postoperative deaths.

## Discussion

The PRODIGE-3 trial addresses the optimal regimen for the neoadjuvant treatment of locally advanced esophageal carcinoma. Despite of a strong rational, our study failed to improve the disease outcome, particularly the pCR rate. In the phase II part, the primary goal was to increase the pCR rate > 20 % (with 25 % expected). Our findings showed a pCR rate of 18.5 %, including only 11.1 % ypT0N0, compared to similar approaches that exhibited pCR rate around 30 % [13]. The toxicity of this combination led to a suboptimal chemotherapy dose-intensity which could explain these disappointing results. Indeed, the phase I study established a RD of 5FU 500 mg/m^2^ plus CDDP 40 mg/m^2^ combined with a fixed standard dose of cetuximab and a concurrent preoperative RT of 45 Gy (25 fractions over 5 weeks).

In the phase I part, one DLT occurred at level −1 (grade anaphylactic reaction), leading to the inclusion of three additional patients. Four DLT out of 6 patients occurred at level 0 (two grade 3 esophagitis and two severe postoperative complications) leading to the choice of dose level −1 as the RD. In the phase II part, while the rate of preoperative complications remained low, the incidence of unexpected postoperative complications, either surgical or medical, was higher than those described elsewhere [2], [3], [4]. Notably, the PRODIGE-3 trial was one of the first to consider postoperative morbidity/mortality in addition to preoperative events in this setting.

The mortality rate reached 12.5 %, approximately twice what was expected [2], [3], [4]. However, only half of the patients died from disease progression.

One could hypothesize that the high rate of postoperative complications was related to a lack of experience among surgical teams, although all the surgical centers involved in this study were high-volume centers. Moreover, based on available evidence, the addition of esophagectomy to C-RT in locally advanced esophageal SCC provides little or no difference in overall survival and may be associated with higher treatment-related mortality [14]. The addition of esophagectomy probably delays locoregional relapse, although this endpoint was not well defined.

In terms of RT, the delivered dose and its fractioning were in agreement with classical regimens in this setting [15]. However, the high rate of postoperative complications might raise questions about target volumes and quality control in the PRODIGE-3 trial.

Another hypothesis could be related to the neoadjuvant treatment. Although Sjoquist *et al.* demonstrated that preoperative C-RT provided a survival benefit over surgery alone [2], the optimal preoperative C-RT regimen in combination with cetuximab was not standardized when the PRODIGE-3 trial was initiated. In our trial, the starting dose level 0 (5FU 600 mg/m^2^, CDDP 60 mg/m^2^) was selected to mimic the dose-intensity of 5FU and CDDP reported in the RTOG trial with definitive chemoradiation. The 5FU/CDDP regimen could be an inadequate chemotherapy combination with cetuximab, as recently described in a similar trial for esophageal carcinoma, where half of the patients developed grade 4 toxicities [16].

Unexpectedly, the addition of cetuximab to a standard chemotherapy did not improve the pCR rate. This could be due to the low chemotherapy dose-intensity compared to the dose-intensity used in other study. The addition of EGFR inhibitors, like Cetuximab, to C-RT may not improve survival and seems to worsen toxicity [16], [17], [18], [19]. Some randomized studies evaluated C-RT combined with cetuximab in non-resectable and non-metastatic esophageal carcinoma. The SCOPE-1 trial investigated the efficacy of adding cetuximab to CDDP-capecitabine compared with C-RT alone [19]. In this study, the C-RT plus cetuximab group had a shorter median overall survival than the C-RT alone group, and the rate of grade 3–4 non-hematological toxicities was higher in the cetuximab arm. The RTOG-0436 trial compared C-RT (CDDP-paclitaxel), with or without cetuximab, showing that the addition of cetuximab did not provide a survival benefit [17].

A phase I/II study (SAKK 75/06) investigated the interest of cetuximab plus CT (CDDP-docetaxel) in the preoperative setting [11]. The pCR rate was 32 % without treatment-related deaths, but postoperative complications were not explored. Notably, this regimen did not include 5FU, which could be poorly tolerated. Indeed, a study evaluating preoperative C-RT with oxaliplatin, 5FU and cetuximab was prematurely stopped due to an increase in postoperative deaths [16]. As demonstrated in head and neck cancer and lung cancer, combining cetuximab and (chemo)radiotherapy is not advisable [20].

Furthermore, the choice of the Mandard classification to assess pCR [12] could be inappropriate because it only considers tumor regression compared with pathological TNM [21].

Various clinical, radiological, serological and potential molecular markers have been studied. Recently, the use of immunotherapy after R-CT and surgery has shown to be sufficient reliability to be used in daily practice [22]. For patients with Deficient Mismatch Repair/Microsatellite Instability-High, Nivolumab and ipilimumab-based neoadjuvant therapy is feasible and has shown excellent results [23]. Certainly, more understanding of the molecular basis for response to chemotherapy/radiotherapy is needed to tailor and individualize patient treatment [24].

## Conclusions

The addition of cetuximab to a preoperative C-RT with the 5FU/CDDP combination has been shown to be toxic, without increasing pCR rates, which raises questions about the further development of this combination.

## Ethics approval and consent to participate

The PRODIGE-3 protocol was reviewed and approved by the French Ethics Committee/Institutional Review Board (Sud-Mediterranée II, Marseille, France, December 1st 2006). Written informed consent was obtained before randomization.

## Consent for publication

Not applicable.

## Availability of data and material

The database was available in the Fédération Francophone de Cancérologie Digestive (FFCD), Dijon, France. Phone: +33 (0)3 80 38 13 14.

## Funding

Merck Serono provided cetuximab.

The collection, analysis, and interpretation of data were performed by the Fédération Francophone de Cancérologie Digestive (FFCD), Dijon, France.

## Authors’ contribution

BDR: Study design, data interpretation, manuscript drafting, critical revisions of manuscript for important intellectual content, final approval of version to be published, and agreement to be accountable for all aspects of the work.

GP: Study design, data interpretation, critical revisions of manuscript for important intellectual content, and final approval of version to be published.

MJ: Study design, data interpretation, critical revisions of manuscript for important intellectual content, and final approval of version to be published.

KLM: Data interpretation, critical revisions of manuscript for important intellectual content, and final approval of version to be published.

AA: Study design, data interpretation, critical revisions of manuscript for important intellectual content, and final approval of version to be published.

TM: Study design, data interpretation, critical revisions of manuscript for important intellectual content, and final approval of version to be published.

XBDJ: Study design, data interpretation, critical revisions of manuscript for important intellectual content, and final be published.

CP: Study design, data interpretation, critical revisions of manuscript for important intellectual content, and final approval of version to be published.

JBM: Study design, data interpretation, critical revisions of manuscript for important intellectual content, and final approval of version to be published.

TA: Study design, data interpretation, critical revisions of manuscript for important intellectual content, and final approval of version to be published.

RG: Study design, data interpretation, critical revisions of manuscript for important intellectual content, and final approval of version to be published.

VV: Study design, data interpretation, critical revisions of manuscript for important intellectual content, and final approval of version to be published.

CL: Study design, data interpretation, critical revisions of manuscript for important intellectual content, and final approval of version to be published.

LD: Study design, data interpretation, manuscript drafting, critical revisions of manuscript for important intellectual content, final approval of version to be published, and agreement to be accountable for all aspects of the work.

All authors read and approved the final manuscript.

## Declaration of Competing Interest

The authors declare the following financial interests/personal relationships which may be considered as potential competing interests: TA declares Conference for Pfizer, Roche, Sanofi, Léo Pharma, Amgen, BMS, Servier, Shire, Ipsen ; Board for Pierre Fabre Ipsen, HalioDX, BMS ; Travels and congress for Ipsen, Novartis, Roche, Hospira : Research grant from Novartis. RG declares travels grants and congress registration from Merck.
